# Clinical presentations of atypical forms of Alzheimer's Disease and its correlates with functional imaging: the importance of early detection, a series of cases

**DOI:** 10.1002/alz.085078

**Published:** 2025-01-03

**Authors:** Francesca Mariani

**Affiliations:** ^1^ Hospital de Clínicas, Montevideo, Montevideo Uruguay

## Abstract

**Background:**

The prevalence of Dementia in Latin America countries is growing and early presentations of Alzheimer´s Diseases with atypical forms are even more frequently. As the clinical presentation of these variants shows an overlap with other dementia disorders, the differential diagnosis is often challenging. We presented three cases of atypical forms of AD who count with cognitive assessment and pet imaging confirmation in order to being able to discuss the most important points of clinical assessment in every case.

**Method:**

Case 1: 60‐year‐old woman with a 2‐year history of progressive anterograde memory deficit, that evolved with a typical corticobasal syndrome with extrapyramidal signs and left superior limb dystonia. Case 2: 65‐year‐old woman with a 1‐year history of progressive visual loss, difficulty in stimuli detection that evolved with memory loss and Anosognosia. Case 3: 68‐year‐old man with a 2‐year history of speech difficulties with reduced fluency and verbal initiative, compromise in naming, repetition and understanding of syntactic relationships. Associate alexia and agraphia. In evolution adds executive and memory impairment, visuoconstructional deficits and ideomotriz apraxia. The three cases count with 18F‐FDG and 11C‐PIB PET/ CT studies supported the diagnosis of Alzheimer’s disease.

**Result:**

This are three cases of atypical presentations of AD with imaging biomarkers confirmation and its particular cognitive assessment characteristics. In two cases, the beginning of the functional history is what marks the differential, in the third case, the evolution is what marks the characteristics of the disease.

**Conclusion:**

Even when we have imaging studies, and especially when we do not have them, which is the majority of cases in Latin America, the clinical examination and in‐depth history are crucial to detect early symptoms. These symptoms could constitute the key elements for an adequate differential diagnosis. Likewise, atypical cases invite us clinicians to carry out rigorous and timely follow‐up, without taking the clinical presentation of our patients for granted. The advancement of technology and imaging biomarkers has changed the way we do clinical work, but we must not forget the value of the latter.

